# Quantitative Digitography Measures Fine Motor Disturbances in Chronically Treated HIV Similar to Parkinson’s Disease

**DOI:** 10.3389/fnagi.2020.539598

**Published:** 2020-10-07

**Authors:** Varsha Prabhakar, Talora Martin, Eva M. Müller-Oehring, Ryan Goodcase, Tilman Schulte, Kathleen L. Poston, Helen M. Brontë-Stewart

**Affiliations:** ^1^Department of Neurology and Neurological Sciences, Stanford University School of Medicine, Stanford, CA, United States; ^2^Leonard M. Miller School of Medicine, University of Miami, Miami, FL, United States; ^3^School of Medicine, Case Western Reserve University, Cleveland, OH, United States; ^4^Center for Health Sciences, Biosciences Division, SRI International, Menlo Park, CA, United States; ^5^Department of Psychiatry and Behavioral Sciences, Stanford University School of Medicine, Stanford, CA, United States; ^6^Department of Clinical Psychology, Palo Alto University, Palo Alto, CA, United States; ^7^Department of Neurosurgery, Stanford University School of Medicine, Stanford, CA, United States

**Keywords:** HIV—human immunodeficiency virus, Parkinson’s disease, motor control, aging, fine motor activities

## Abstract

**Introduction**: Motor and cognitive deficits were compared in aging, chronically treated human immunodeficiency virus (HIV) people, people with mild-to-moderate stage Parkinson’s disease (PD), and healthy controls.

**Methods**: Groups consisted of 36 people with PD, 28 with HIV infection, and 28 healthy controls. Motor function was assessed with the Unified Parkinson’s Disease Rating Scale (MDS-UPDRS-III) and a rapid alternating finger tapping (RAFT) task on an engineered keyboard known as Quantitative Digitography (QDG). Executive function, verbal memory, and visuospatial processing were assessed using standard neuropsychological tests.

**Results**: HIV demonstrated RAFT deficits similar to PD such as reduced amplitude (*P* = 0.023) and greater amplitude variability (*P* = 0.019) in the index finger when compared to controls. This fine motor disturbance correlated with HIV’s immune health, measured by their CD4^+^ T cell count (*P* < 0.01). The UPDRS did not yield motor differences between HIV and controls. Executive function and verbal memory were impaired in HIV (*P* = 0.006, *P* = 0.016, respectively), but not in PD; visuospatial processing was similarly impaired in HIV and PD (*P* < 0.05) although motor deficits predominated in PD.

**Conclusions**: Fine motor bradykinesia measured quantitatively by QDG RAFT holds promise as a marker of motor decline related to current immune health in aging HIV patients and may be useful in longitudinal studies regarding mechanisms of immunosenescence vs. potential toxicity of combination antiretroviral therapy (cART) in this population. Additionally, motor and cognitive networks in HIV may be affected differently as the disease progresses as observed in the differential patterns of impairment between HIV and PD, providing insight into the mechanisms of brain deterioration in HIV.

## Introduction

Human immunodeficiency virus (HIV) is no longer a terminal illness for most of the population due to the success of combination antiretroviral therapy (cART), which suppresses HIV replication. By 2030, over 70% of the HIV population will be 50 years or older, and chronically treated HIV patients are expected to reach life expectancies similar to the general population (Greene et al., 2013; Smit et al., 2015; McLaurin et al., 2019). Although cART therapy decreased the incidence of motor deficits in HIV, there is evidence that chronic HIV and cART facilitates alpha-synuclein deposition in the substantia nigra and basal ganglia atrophy, suggesting that people who are aging with chronically treated HIV may be at risk of developing motor deficits similar to those encountered in people with PD (Khanlou et al., 2009; Tesic et al., 2018). Furthermore, studies show that viral protein expression itself predominates in the basal ganglia which supports a possible expression of Parkinsonism symptoms as the disease progresses (Wiley et al., 1998; Schier et al., 2017). Also, mild-to-moderate forms of HIV-associated cognitive impairment affect up to 50% of people with HIV (Sacktor et al., 2001; Woods et al., 2009; Smail and Brew, 2018). This incidence persisted in people on chronic cART compared to those who were not on cART (Grant et al., 2005; Mateen et al., 2012). It is unclear how overall patterns of cognitive and motor impairment differ in HIV compared to PD.

Early detection of motor and/or cognitive deficits in aging, treated HIV individuals may lead to improved clinical and behavioral interventions. Quantitative Digitography (QDG) is a novel repetitive alternating finger tapping task (RAFT) performed on an engineered keyboard that shows sensitivity to motor deficits in very early stage untreated PD (Brontë-Stewart et al., 2000; Taylor Tavares et al., 2005; Koop et al., 2008). The QDG RAFT task is employed in this study to explore its use as a quantifiable measurement of fine motor deficits in HIV as well.

In this study, we compared the patterns and degree of motor and cognitive function in a group of aging, chronically treated HIV, to a group of mild-to-moderate stage PD, and healthy controls. We hypothesized that HIV and PD would show similar types of motor and cognitive deficits but with varying dominance when compared to healthy controls.

## Materials and Methods

### Participants

Thirty-six people (21 males) with mild-to-moderate PD, 28 people (18 males) with chronically treated HIV infection, and 28 (13 males) healthy controls (C) participated in the study. All participants were between the ages of 45 and 80 years, had at least 8 years of education, and had no history of psychiatric diseases (i.e., schizophrenia or bipolar I disorder), neurological illnesses other than PD, medical conditions potentially affecting the CNS other than HIV, or MRI contraindications. No subjects were excluded based on cognitive severity measured by the Dementia Rating Scale. PD subjects recruited from community clinics and support groups were included if they had a disease duration greater than or equal to 2 years, received an average levodopa equivalent daily dose (LEDD) of 587 mg (median 535 mg, range 100–1,440 mg), had levodopa-responsive motor signs, and were able to walk unassisted (Hoehn and Yahr stage < V) when off medication. Medication-induced dyskinesias may have been present in the on- medication state in PD subjects on higher doses of medication. HIV subjects were included if they had a seropositive HIV test and CD4^+^ T cell counts greater than 200 cell/mm^3^ at the time of the study. Out of 28 HIV participants, 17 met the criteria for AIDS (CD4^+^ nadir < 200 cell/mm^3^ and/or AIDS-defining event during their disease history). All HIV participants were aviremic (no detectable viral loads), were on stable antiretroviral therapy, and had not changed their medication regimen in the past 2 months before the study. All HIV participants were on a continuous regimen of combination antiretroviral treatment (cART) that included two or more nucleoside reverse transcriptase inhibitors (NRTIs) combined with integrase inhibitors (II; NRTIs and II), protease inhibitors (NRTIs and PI), non-nucleoside reverse transcriptase inhibitors (NRTIs and NNRTI), or a combination thereof (NRTIs, NNRTI, and II; NRTIs, II, and PI). Control subjects were included if they had a normal neurological examination performed by a licensed neurologist and tested negative for HIV and Hepatitis C and were excluded if they scored ≥10 on the Movement Disorder Society–Unified Parkinson’s Disease Rating Scale part III motor scale (MDS–UPDRS-III) to ensure a healthy, non-Parkinsonian group for accurate comparison (Goetz et al., 2007). No subjects had sensory neuropathy in their upper extremities as measured in the neurological exam.

### Experimental Protocol

After being screened for eligibility, participants were assessed with the MDS-UPDRS-III by a certified rater from which subscores of axial disability (items 1–3 and 9–14), tremor (items 15–18), bradykinesia (items 4–8), and rigidity (item 3) were extracted. Quantitative measurements of fine motor control were measured using a validated RAFT on an engineered keyboard (QDG; Brontë-Stewart et al., 2000; Taylor Tavares et al., 2005; Trager et al., 2015). Cognition was assessed using a comprehensive neuropsychological battery. PD subjects were on dopaminergic medication during cognitive testing to minimize the influence of motor impairment on their performance whereas motor testing was performed both on (PD-on) and off (PD-off) dopaminergic medication for levodopa-responsive motor signs. Off medication was defined as at least 48 h off extended-release dopamine agonists, at least 24 h off long-acting medication, and at least 12 h off short-acting medication.

### Quantitative Measurement of Movement With RAFT

**Figure 1:**
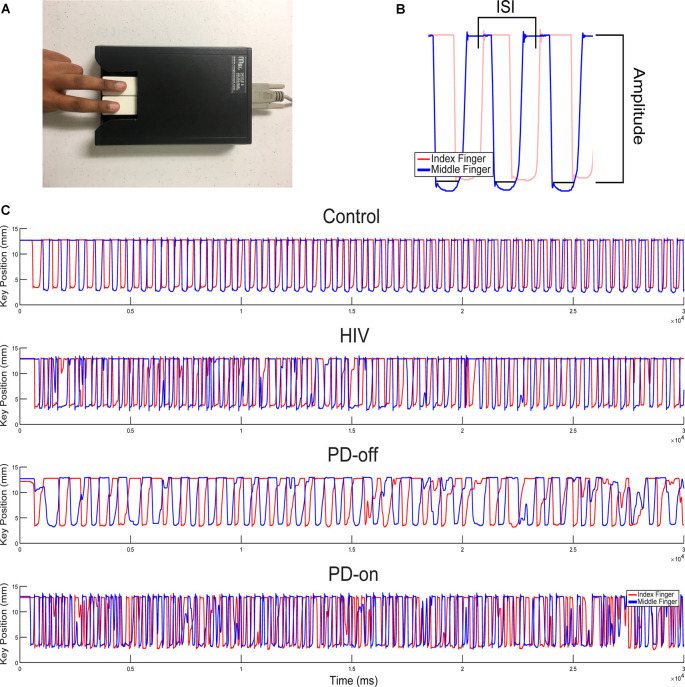
Quantitative digitography (QDG) and sample rapid alternating finger tapping (RAFT) traces. **(A)** Index and middle finger placement on the engineered keyboard (QDG). **(B)** Three cycles of RAFT demonstrating the ISI (inter-strike-interval) and peak-to-peak amplitude of a finger strike cycle. **(C)** Representative 30-s RAFT traces from the MN (More affected, Non-dominant) hand of control, HIV, and PD off and on dopaminergic medication (highest to lowest panels).

Fine motor control in the hands was measured using the QDG RAFT task that has been validated with the MDS-UPDRS-III (Brontë-Stewart et al., 2000; Taylor Tavares et al., 2005; Koop et al., 2008; Trager et al., 2015). Subjects placed their index and middle fingers on each key (Figure 1A) and were instructed to tap their fingers in an alternating pattern as fast and as regularly as possible for 30 s. Visual and auditory feedback was removed by having subjects close their eyes and wear headphones playing “white noise” to mask the sound of their finger strikes. Both hands were measured.

### RAFT Data Acquisition and Processing

A customized algorithm, written in MATLAB (The Mathworks, Inc., Natick, MA, USA) was used to acquire four measurements from the QDG RAFT output (Figure 1B): frequency, frequency variability (rhythmicity), amplitude, and amplitude variability. Frequency (Hz) is the inverse of the average inter-strike-interval (ISI) or the time to complete one cycle of finger tapping in seconds. Rhythmicity is the coefficient of variation of the ISI (CV_ISI_). Amplitude is the distance (mm) the key traveled during an entire cycle of tapping from pressing down to release and amplitude variability is its coefficient of variation (CV_AMP_). Poor RAFT performance is defined as having a lower frequency, higher frequency variability, lower amplitude, and higher amplitude variability. Progressive bradykinesia, or progressive slowing of movement, as found through RAFT performance is defined as the increase of ISI and/or decrease of amplitude values over time (Brontë-Stewart et al., 2000).

Each hand was identified as either the MN (the more affected side in PD subjects; Non-dominant hand in HIV and C subjects) or LD (the less affected side in PD subjects; Dominant hand in HIV and C subjects) hand; this pairing was used to avoid biasing the results between the groups towards greater difference. The more affected side in PD subjects was the side of their first self-reported symptom. If the first symptom was bilateral (such as in gait), it was determined by the MDS-UPDRS-III score for each side (Louie et al., 2009). The results in this article focus on the MN hand to examine the side with the greatest impairment in PD and the non-dominant side in the HIV and control groups, consistent with neuroimaging evidence suggesting sensitivity of the non-dominant hand, but not the dominant hand, for motor output aberration with older age (Schulte et al., 2013) and disease (Schulte et al., 2010).

### Cognitive Assessments

Executive functioning was measured with the Golden Stroop Test (Stroop, 1935) and Digit Span Backward Test (DSB; Wechsler, 1987). The Golden Stroop color word (CW) score and DSB scores reported are the number of correct responses. Verbal memory was measured with the California Verbal Learning Test (CVLT; Woods et al., 2006). Two variables were analyzed: total immediate recall (number of words recalled over five trials) and long delay free recall (number of words recalled from the initial word list 20 min after a second-word list is presented). Visuospatial processing was measured with the Judgment of Line Orientation (JLO; Benton et al., 1983). All tests were administered and scored according to standardized instructions.

### Statistics

To test differences between the three groups, univariate and multivariate analysis of covariance (ANCOVA and MANCOVA), corrected for age and education, were conducted using SPSS (v.25, Armonk, NY, USA). *Post hoc* least significant differences (LSD) tests were used to examine the individual differences between group means. Based on the directed hypothesis that people with worse immune health (CD4^+^ T cell count, CD4^+^ nadir) would have worse motor and cognitive performance, partial correlations were tested for 1-tailed significance, and controlled for the effect of age. An α level of *P* < 0.05, two-tailed, was used for all other statistical tests.

### Standard Protocol Approvals, Registrations, and Patient Consents

This study was approved by the Stanford School of Medicine and Stanford Research Institute Institutional Review Boards. All subjects gave written informed consent before any study-related testing took place.

### Data Availability Policy

Anonymized data will be shared by request from any qualified investigator.

## Results

The average disease duration for the PD group was 4.6 ± 2.9 years and the HIV group was 24.2 ± 8.0 years. The PD group had an average Hoehn and Yahr stage of 2.1, demonstrating mild-to-moderate stage PD characterized by bilateral symptoms without postural instability. The HIV group’s mean CD4^+^ T cell count at the time of the study was 769.48 cells/mm^3^ and the average CD4^+^ nadir was 186.96 cells/mm^3^. The majority race of the sample was white (C: *n* = 17, HIV: *n* = 16, PD: *n* = 33). Nine HIV and five C were black. Two C were Asian. One HIV and two C were Pacific Islanders. Three PD, two HIV, and two C identified as “other” or “more than one race.” *Post hoc* LSD tests showed that the PD group (65.5 ± 7.7) was older in years than the HIV (59.8 ± 6.9, *P* = 0.004) and C groups (61.1 ± 8.6, *P* = 0.027); the HIV group was of a similar age to the C group. The HIV group had fewer years of education (14.4 ± 2.3) than both the PD (16.7 ± 2.1, *P* < 0.0001) and C groups (16.0 ± 2.3, *P* = 0.007). Finally, the Beck Depression Inventory scores of all the groups lay in the minimal range of 0–13 (C: 2.4 ± 3.4, HIV: 9.5 ± 7.9, PD: 6.4 ± 4.6).

The total UPDRS-III scores and axial, bradykinesia, rigidity, and tremor subscores showed a significant multivariate effect (MANCOVA, Pillai’s Trace *F*_(10,168)_ = 7.806, *P* < 0.0001), controlled for age and education. All univariate tests for UPDRS total and subscores were significant (all *F*_(2,87)_ > 26.1, *P* = 0.0001). *Post hoc* LSD analyses showed higher scores in PD-off, meaning off levodopa medication (20.6 ± 10.1) compared to HIV (4.3 ± 4.3) and C (1.2 ± 1.8, all *P* = 0.0001). Similar effects were found in PD-on levodopa (total UPDRS score: 15.9 ± 8.0). The HIV group did not differ from the C group but tended to be more bradykinetic (bradykinesia subscores for HIV: 1.9 ± 2.3 and C: 0.6 ± 0.9; *P* = 0.065). An ANCOVA adding race as a between-subjects variable (racial groups: white, black, other) showed that group differences in UPDRS-III scores and axial, bradykinesia, rigidity, and tremor subscores did not differ depending on race (Pillai’s Trace *F*_(10,158)_ = 0.262, *P* = 0.99), and group-by-race interactions (Pillai’s Trace *F*_(15,240)_ = 0.435, *P* = 0.97).

### Chronically Treated HIV Exhibits Progressive Bradykinesia and Arrhythmicity of Fine Motor Control

Figures 1A,B demonstrate the RAFT task on an engineered keyboard (QDG) which measured amplitude and inter-strike interval (ISI). Figure 1C demonstrates the performance of RAFT by the MN hand of a representative control (C), HIV, and PD off and on medication, respectively. The C performed regular RAFT with full amplitude depression of the key and a regular ISI. The HIV’s ISI and amplitude appeared similar but more irregular than C. The PD-off ISI was longer (lower frequency) and the amplitude of tapping was variable. Observation of the trace shows that the ISI increased and amplitude decreased over time, demonstrating progressive bradykinesia. The same PD person on medication (PD-on) exhibited higher ISI compared to PD-off but frequency and amplitude were still irregular.

The group statistics supported the individual traces in Figure 1C: the multivariate effect for RAFT measures was significant (MANCOVA, Pillai’s Trace *F*_(8,162)_ = 2.652, *P* = 0.009). Univariate tests showed group differences between PD-off, HIV, and C in amplitude (*F*_(2,83)_ = 5.862, *P* = 0.004), in the variability of the ISI or “rhythmicity” (CV_ISI_, *F*_(2,83)_ = 3.977, *P* = 0.022), and in the variability of amplitude (CV_AMP_, *F*_(2,83)_ = 4.567, *P* = 0.013) for the MN index finger.

**Figure 2:**
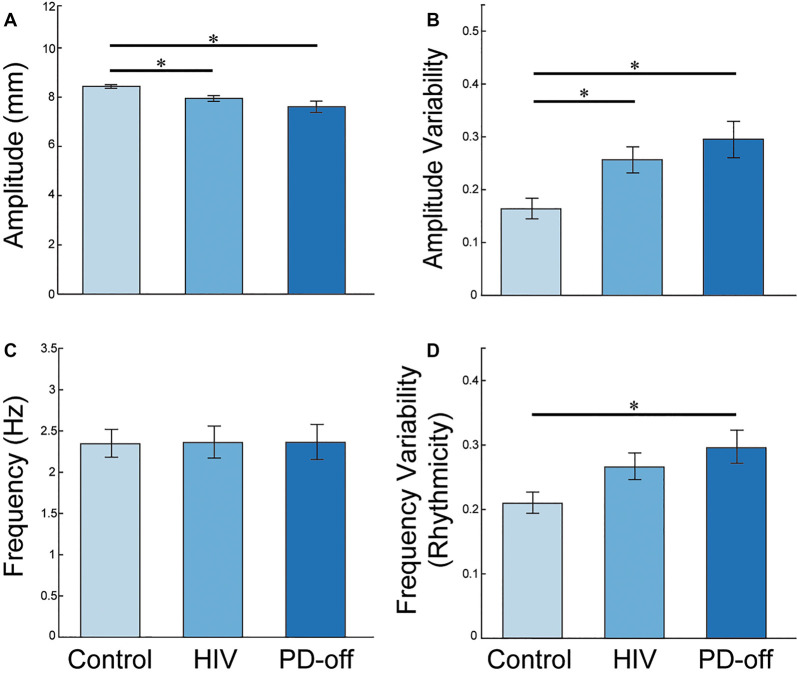
MN Index Finger RAFT Measures. Post-hoc least significant differences (LSD) group comparisons of RAFT metrics in the MN index finger (the more affected side in PD subjects; Non-dominant hand in HIV and C subjects): **(A)** amplitude (distance the key travelled during a cycle of tapping from pressing down to release), **(B)** amplitude variability (CV_AMP_). PD-off = off medication, **(C)** frequency [inverse of the average inter-strike-interval (ISI) or time to complete one cycle of finger tapping in seconds], and **(D)** amplitude variability (CV_AMP_). PD-off = off medication. Error bars indicate SEM. *Indicates significance, *P* < 0.05.

*Post hoc* LSD analyses demonstrated reduced amplitude and greater amplitude variability in PD-off (*P* = 0.002 and *P* = 0.009, respectively) and HIV (*P* = 0.023 and *P* = 0.019, respectively) compared to C. PD-off and HIV were similar in these kinematic parameters, Figures 2A,B. PD-off were more arrhythmic than C (*P* = 0.008); HIV tended to be more arrhythmic than C and more rhythmic than PD but did not significantly differ between both groups, Figure 2D. Average tapping frequency did not vary among groups, Figure 2C. All of the above effects were similarly found in the PD-on index finger, data not shown. The MN middle finger and MN average (between the index and middle finger) value showed higher frequency variability, lower amplitude, and higher amplitude variability in PD-off compared to HIV and C (*P* < 0.05), and no difference between HIV and C. Results remained significant adding cognitive variables as covariates to the model (Pillai’s Trace *F*_(8,148)_ = 2.364, *P* = 0.020; amplitude (*F*_(2,76)_ = 4.695, *P* = 0.012; CV_ISI_, *F*_(2,78)_ = 3.089, *P* = 0.05, CV_AMP_, *F*_(2,78)_ = 3.690, *P* = 0.03).

We repeated the analyses for the dominant/less affected hand, which confirmed our findings: multivariate effect for RAFT measures was significant (MANCOVA, Pillai’s Trace *F*_(8,158)_ = 2.436, *P* = 0.016). Univariate tests for the dominant hand showed group differences between PD-off, HIV and C in amplitude (*F*_(2,81)_ = 4.592, *P* = 0.013), in variability of the ISI or “rhythmicity” (CV_ISI_, *F*_(2,81)_ = 5.944, *P* = 0.004), and in variability of amplitude (CV_AMP_, *F*_(2,81)_ = 4.652, *P* = 0.012) for the LD index finger. Again, no group difference was observed for frequency (*F*_(2,81)_ = 1.201, *P* = 0.306). Combined MN and LD index fingers also confirmed the group differences for each RAFT measure: amplitude (*F*_(2,77)_ = 7.646, *P* = 0.001; HIV vs. C: *P* = 0.039, PD vs. C: *P* < 0.0001), rhythmicity (CV_ISI_, *F*_(2,77)_ = 8.049, *P* = 0.001; PD vs. C: *P* < 0.0001, PD vs. HIV: *P* = 0.028), and amplitude variability (CV_AMP_, *F*_(2,77)_ = 7.209, *P* = 0.001; HIV vs. C: *P* = 0.020, PD vs. C: *P* < 0.0001), and no effect for frequency (*F*_(2,77)_ = 1.854, *P* = 0.164). Race did not significantly affect QDG scores (Pillai’s Trace *F*_(8,152)_ = 0.376, *P* = 0.93) or contribute to group differences in QDG scores (group-by-race interaction Pillai’s Trace *F*_(12,231)_ = 0.466, *P* = 0.93). Directly comparing non-dominant and dominant hand performances in a mixed measure ANCOVA revealed no significant effects for hand (dominant, non-dominant) over all study participants (amplitude, *F*_(1,77)_ = 0.459, *P* = 0.500; CV_ISI_, *F*_(1,77)_ = 0.222, *P* = 0.639; CV_AMP_, *F*_(1,77)_ = 0.384, *P* = 0.537; frequency, *F*_(2,81)_ = 1.201, *P* = 0.306). No significant group-by-hand interactions were forthcoming (amplitude, *F*_(2,77)_ = 0.821, *P* = 0.444; CV_ISI_, *F*_(2,77)_ = 0.357, *P* = 0.701; CV_AMP_, *F*_(2,77)_ = 0.320, *P* = 0.727; frequency, *F*_(2,81)_ = 1.201, *P* = 0.306).

### Progressive Bradykinesia and Arrhythmicity in Chronically Treated HIV Subjects Correlates with CD4^+^ T Cell Count

**Figure 3:**
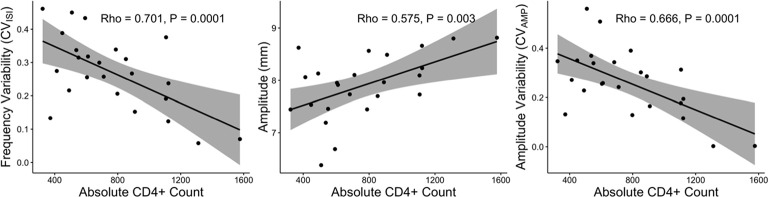
RAFT Measures and CD4^+^ T cell count. Correlations between impaired RAFT metrics (frequency variability, amplitude and amplitude variability; left to right panels) and CD4^+^ count in the HIV group.

There were robust correlations between the HIV kinematics of progressive bradykinesia and their CD4^+^ T cell count, which is a marker of immune health in HIV, Figure 3. Lower (worse) CD4^+^ T cell count correlated with greater arrhythmicity, lower amplitude and greater amplitude variability (controlling for age using partial correlations: CV_ISI_: *r* = −0.645, *P* = 0.001, Figure 3A; Amplitude: *r* = 0.512, *P* = 0.01, Figure 3B; CV_AMP_: *r* = −0.599, *P* = 0.003, Figure 3C).

The MN average and MN middle finger alone values for amplitude, CV_ISI_, and CV_AMP_ also correlated with CD4^+^ T cell count (*P* < 0.05, one-tailed). The LD hand RAFT measures did not correlate with the CD4^+^ T cell count.

### Cognitive Deficits in Chronically Treated HIV Affects More Domains Than in Mild-to-Moderate PD

Univariate tests controlled for age and education showed group differences in executive function (Stroop CW: *F*_(2,86)_ = 4.693, *P* = 0.012; DSB: *F*_(2,86)_ = 4.168, *P* = 0.019), verbal memory (CVLT total immediate recall: *F*_(2,87)_ = 1.443, *P* = 0.242; CVLT long delay: *F*_(2,87)_ = 3.201, *P* = 0.046) and visuospatial processing (*F*_(2,87)_ = 3.822, *P* = 0.026).

*Post hoc* LSD analyses showed that HIV performed worse in all three domains compared to C (Stroop CW: *P* = 0.003 and DSB: *P* = 0.006; CVLT long delay: *P* = 0.016; JLO: *P* = 0.040). Mild-to-moderate PD had descriptively lower scores than C, but scores did not differ statistically for executive function (with a trend towards worse performance in the DSB task, *P* = 0.087) and verbal memory (*P* > 0.05). PD demonstrated lower performance scores in visuospatial processing compared to C (*P* = 0.013), which was similar to HIV (Table 1).

**Table 1:** Raw scores and group differences for cognitive tests.

	Controls M (SD) *[95% CI]*	HIV M (SD) *[95% CI]*	PD-on M (SD) *[95% CI]*	*P*-value^a^	*Post hoc* LSD tests groups (*P-value*)
**Executive functioning**
Stroop CW	41.68 (10.28) *[37.69, 45.66]*	35.19 (9.32) *[31.50, 38.87]*	36.72 (9.16) *[33.62, 39.82]*	0.012	PD = C (0.284) PD = H (0.051) H < C (0.003)
DSB	8.21 (2.30) *[7.32, 9.11]*	6.07 (2.73) *[5.06, 7.24]*	7.09 (2.24) *[6.32, 7.86]*	0.019	PD = C (0.087) PD = H (0.248) H < C (0.006)
**Verbal memory**
CVLT total immediate recall	50.96 (10.28) *[46.98, 54.95]*	44.36 (12.06) *[39.68, 49.03]*	47.25 (11.70) *[43.29, 51.21]*	ns (0.242)	PD = C (0.202) PD = H (0.735) H = C (0.123)
CVLT Long Delay	12.14 (3.22) *[10.90, 13.39]*	9.32 (3.94) *[7.80, 10.85]*	10.64 (3.50) *[9.46, 11.82]*	0.046	PD = C (0.118) PD = H (0.344) H < C (0.016)
**Visuospatial processing**
JLO	26.64 (3.63) *[25.23, 28.05]*	23.43 (4.60) *[21.65, 25.21]*	24.11 (4.32) *[22.65, 25.57]*	0.026	PD < C (0.013) PD = H (0.772) H < C (0.040)

*Stroop CW, Golden Stroop Test color word score; DSB, Digit Span Backwards test; CVLT, California Verbal Learning Test; JLO, Judgment of Line Orientation test. ^a^Multivariate and univariate significance levels*.

Group differences for cognitive scores remained significant when race was added as a between-subjects variable (racial groups: white, black, other). A significant main effect for race was observed for some executive function tests (Stroop CW *F*_(2,81)_ = 4.954, *P* = 0.009) but not others (DSB: *F*_(2,81)_ = 2,307, *P* = 0.106). Race did not significantly affect verbal memory (CVLT total immediate recall: *F*_(2,82)_ = 1.846, *P* = 0.164; CVLT long delay: *F*_(2,82)_ = 1.926, *P* = 0.152) and visuospatial processing (JLO: *F*_(2,82)_ = 1.846, *P* = 0.164). Group effects on cognitive scores were independent of race effects (no group-by-race interaction; all *P’s* > 0.05).

Cognitive performance in HIV individuals did not differ depending on an AIDS diagnosis (MANOVA Pillai’s Trace *F*_(5, 20)_ = 0.58, *P* = 0.718; univariate tests: Stroop CW *F*_(1,24)_ = 0.441, *P* = 0.513; DSB: *F*_(1,24)_ = 0.031, *P* = 0.863; CVLT total immediate recall: *F*_(1,24)_ = 0.074, *P* = 0.788; CVLT long delay: *F*_(1,24)_ = 0.217, *P* = 0.645; JLO: *F*_(1,24)_ = 0.026, *P* = 0.874).

### No Relationship Exists Between RAFT and Cognitive Ability and CD4^+^ T Cell Count

No correlation existed between the RAFT values and cognitive measures or between cognitive measures and current immune health (CD4^+^ T cell count) and past HIV disease severity (CD4^+^ nadir) in the HIV group (all *P*’s > 0.05).

## Discussion

This study demonstrated that people aging with chronically treated HIV infection display impairment in fine motor control similar to that seen in Parkinson’s disease, and poorer performance in cognitive domains such as executive, visuospatial and verbal memory compared to healthy controls of a similar age. Fine motor control impairment in the HIV group was measured through a quantitative RAFT task on an engineered keyboard and correlated with the group’s immune health, measured by CD4^+^ T cell count. This impairment and correlation were not found when the standard clinical MDS-UPDRS-III was used. Finally, cognitive and motor disturbances in the HIV group were similar, but affected in a different pattern than a group with moderate PD: compromised cognitive functioning, with mild motor deficits, predominated in HIV participants, while motor (and visuospatial) impairments predominated in mild-to-moderate stage PD patients, shedding insight into the patterns of disease mechanism and progression for both groups.

At the time of testing, the HIV cohort did not include anyone with a CD4^+^ T cell count <200 per mm^3^ and most of the HIV cohort had normal CD4^+^ counts (>500 cells/mm^3^). Thus, despite efficient immunosuppression, they still exhibited poorer motor and cognitive performance compared to healthy controls of a similar age. Also, the HIV group’s arrhythmicity and progressive bradykinesia, measured by QDG-RAFT, inversely correlated with their current immune health, measured by their CD4^+^ T cell count. Despite earlier reports of severe immunosuppression at baseline as an additional risk factor for impairment (Ellis et al., 2011), a relationship between the group’s lowest CD4^+^ counts (nadir/baseline CD4^+^) and RAFT performance was not forthcoming, suggesting that QDG-identified bradykinesia is related to immune changes in the later and controlled stages of HIV in those aging with the disease (Hunt, 2014), rather than the motor deficits observed in early, unmanaged stages (American Academy of Neurology AIDS Task Force, 1991). Longitudinal studies must be completed to determine whether QDG-identified bradykinesia can therefore be used clinically as a marker or predictor of deteriorating immune health in aging, medically managed HIV patients. No correlation existed between cognitive performance and CD4^+^ count (current and nadir).

Before the era of cART, the most common clinical presentations of HIV infection were subcortical motor deficits, slowed cognitive speed, and poor verbal fluency, but after the introduction of cART, it was believed that motor deficits decreased while impairments in memory and executive function became more prominent (Cysique et al., 2004; Robertson et al., 2007; Heaton et al., 2011; Clifford and Ances, 2013). This study demonstrates that aging with chronic HIV infection is associated with subcortical motor deficits that show a pattern similar to those in mild-to-moderate PD which may go undetected with standard clinical scales but are evident with quantitative measures of bradykinesia like QDG.

There has been an ongoing debate whether the underlying mechanisms of cognitive deficits in the chronically treated HIV population are related to the disease itself, the emergence of a neurodegenerative disease like PD, the combined effect of immunosenescence and HIV viral loads, and/or cART toxicity (Smail and Brew, 2018). The data from our study sheds insight into possible mechanisms: the subcortical motor deficits of progressive bradykinesia and arrhythmicity in older, treated HIV people were similar to those seen in mild-to-moderate stage PD people, suggesting that both affect similar sensorimotor circuitry. PD or Parkinsonism might emerge in chronically treated HIV subjects as they age, whether it is due to PD-associated degeneration of, or HIV-associated toxicity to, midbrain dopamine-producing neurons. Successful treatment of Parkinsonian motor deficits with dopamine replacement therapy in an HIV patient supports this mechanistic hypothesis (Devine et al., 2018).

The cognitive performance in the HIV cohort was worse than those exhibited in the mild-to-moderate PD cohort- even after correcting for age and education effects- although they were in domains known to be affected as PD progresses. This raises the possibility that in aging HIV people, there may be additional insults to cortical networks involved in both PD and HIV infection as a result of viral related pathology, cART toxicity, and/or age-related cerebrovascular disease. Neuroinflammation may also play a pathogenetic role in subcortical and cortical networks in both diseases, although at different stages of disease progression. These findings are important in optimizing neurocognitive function in people who can now live longer with chronically treated HIV.

Limitations of this study include the exclusion of more cognitive domains in our group comparisons which could provide additional insight into the underlying mechanisms of poor cognitive performance in older individuals living with chronic HIV infection, compared to age-matched controls, and how it differs from the progression of PD. This study also sheds light on the need for imaging studies to reliably identify structural and functional neural networks that play a role in the progression of aging with HIV and PD. Despite evidence of differences in cognitive aging between racial groups (Weuve et al., 2018), a race-by-diagnosis interaction was not forthcoming, i.e., HIV effects on cognitive performance were not significantly different by race. Although we statistically controlled for age, education and race using covariance methods, future studies with larger samples are needed to elucidate the differences in cognitive compromise in HIV and other neurodegenerative diseases in relation to sociodemographic factors. Lastly, there may be bias towards including subjects in all groups with better cognitive performance than normal since cognitively impaired people may not readily volunteer for clinical research.

## Conclusions

People with chronically treated HIV infection display progressive bradykinesia in fine motor control and worse performance in executive, verbal memory, and visuospatial cognitive measures compared to healthy controls. Progressive bradykinesia and arrhythmicity of a repetitive finger-tapping task on an engineered keyboard (QDG), was inversely related to their CD4^+^ T cell count and may be a marker of immune health even in HIV people with normal CD4^+^ counts (>500 cells/mm^3^). Measures extracted from the RAFT task, therefore, hold promise as markers for motor decline related to current immune health, based on CD4^+^ count, in aging, medically managed HIV patients.

The patterns of cognitive and motor disturbances in the HIV group were similar but inverted compared to a group with moderate PD: the HIV group demonstrated worse cognitive performance in more domains compared to healthy controls and the PD group demonstrated motor impairment in more aspects compared to healthy controls. These results suggest that motor circuitry may be affected by PD-associated degeneration or HIV-associated toxicity to midbrain dopamine-producing neurons, whereas cortical cognitive networks may be more adversely affected in treated HIV due to cART toxicity and/or age-related cerebrovascular disease (Rosenthal and Tyor, 2019). This important distinction has implications for further understanding the mechanisms of brain deterioration in HIV and improving clinical management of the aging, chronically treated HIV patient.

## Data Availability Statement

The datasets generated for this study are available on request to the corresponding author.

## Ethics Statement

The studies involving human participants were reviewed and approved by Stanford School of Medicine Institutional Review Board and Stanford Research Institute Institutional Review Board. The patients/participants provided their written informed consent to participate in this study.

## Author Contributions

VP contributed to data collection, formal analysis, and writing of the manuscript. TM contributed to data collection of the motor measures. EM-O contributed to conceptualization of the study, formal analysis, and writing and review of the manuscript. RG contributed to data collection of the cognitive measures. TS contributed to conceptualization and methodology of the study. KP contributed to conceptualization and draft review. HB-S contributed to the study’s conceptualization and methodology, supervision and coordination, and draft review and editing.

## Conflict of Interest

The authors declare that the research was conducted in the absence of any commercial or financial relationships that could be construed as a potential conflict of interest.
